# Relationship of Meteorological and Air Pollution Parameters with Pneumonia in Elderly Patients

**DOI:** 10.1155/2018/4183203

**Published:** 2018-03-21

**Authors:** Suleyman Serdar Tasci, Cemil Kavalci, Afsin Emre Kayipmaz

**Affiliations:** Emergency Department, Faculty of Medicine, Baskent University, Ankara, Turkey

## Abstract

**Background and Purpose:**

In this study, we aimed to evaluate the relationship between pneumonia and meteorological parameters (temperature, humidity, precipitation, airborne particles, sulfur dioxide (SO_2_), carbon monoxide (CO), nitrogen dioxide (NO_2_), nitrite oxide (NO), and nitric oxide (NOX)) in patients with the diagnosis of pneumonia in the emergency department.

**Methods:**

Our study was performed retrospectively with patients over 65 years of age who were diagnosed with pneumonia. The meteorological variables in the days of diagnosing pneumonia were compared with the meteorological variables in the days without diagnosis of pneumonia. The sociodemographic characteristics, complete blood count of the patients, and meteorological parameters (temperature, humidity, precipitation, airborne particles, SO2, CO, NO2, NO, and NOX) were investigated.

**Results:**

When the temperature was high and low, the number of days consulted due to pneumonia was related to low air temperature (*p* < 0.05). During the periods when PM 10, NO, NO2, NOX, and CO levels were high, the number of days referred for pneumonia was increased (*p* < 0.05).

**Conclusion:**

As a result, climatic (temperature, humidity, pressure levels, rain, etc.) and environmental factors (airborne particles, CO, NO, and NOX) were found to be effective in the number of patients admitted to the hospital due to pneumonia.

## 1. Introduction

Pneumonia, shortly, is the inflammation of lung tissue [1]. It occurs secondary to various microorganisms, mainly bacteria [2]. The annual incidence of community-acquired pneumonia is 2.7–10 per 1000 people and it did not vary much in the last decades [3]. Approximately 250.000 people in Germany are hospitalized in the hospital for pneumonia annually, and it is estimated that twofold this number are treated as outpatients in primary public health centers [4]. Pneumonia is one of the common causes of admission to emergency rooms (ER). The rate of admission to ER due to pneumonia was reported to be 3.7% in a study conducted at Yuzuncu Yil University [5].

The effect of meteorological events on various diseases has been investigated for a while. Their effects on cerebrovascular diseases and coronary artery diseases were examined already [6–8]. The incidence of pneumonia is known to show a seasonal variation. Meteorological variations especially set the ground for tracheitis, bronchitis, bronchial asthma, pneumonia, and other respiratory infections. Liu et al. reported that pneumonia was affected by meteorological factors such as air temperature and humidity [9].

The aim of the present study was to determine the relationship between meteorological and air pollution parameters [temperature, humidity, precipitation, airborne particles, sulfur dioxide (SO_2_), CO2, carbon monoxide (CO), nitrite dioxide (NO_2_), nitrite oxide (NO), and nitric oxide (NOX)] with pneumonia.

## 2. Materials and Methods

Our study was conducted by retrospectively screening the files of the patients aged over 65 years followed up due to pneumonia in the emergency department of Baskent University Ankara Hospital between 01.05.2011 and 01.05.2016 after obtaining the approval from the Ethics Committee.

Baskent University Ankara Hospital is a tertiary foundation university hospital. In our adult emergency department, annual averages of 30000 patients are treated.

The histories, physical examination findings, laboratory findings, and the results of chest X-rays and computed tomography (CT) of all cases were examined. The meteorological variables on the days when the diagnosis of pneumonia was made and meteorological variables on the days when the diagnosis of pneumonia was not made were compared.

Meteorological data were received from the IBM website of https://www.wunderground.com whereas the weather monitoring data were received from the https://www.havaizleme.gov.tr website of the Republic of Turkey Ministry of Environment and Urbanization.

The patients who were admitted to the ER with shortness of breath/respiratory distress and diagnosed with diseases other than pneumonia (pulmonary embolism, COPD exacerbation, etc.), who aged below 65 years and whose files could not be accessed, were excluded from the study.

All statistical analyses were performed by using SPSS version 24.0 for windows. The descriptive data were expressed as mean standard deviation (SD), median, interquartile range (IQR), minimum, maximum, patient number (*n*), and %. The chi-square test was used to compare the categorical variables. Mann-Whitney *U* test was used to compare numerical variables between pneumonia admission and nonadmission days and chi-square test was used to investigate relationship between admission and event (rain). The lag analysis was used to determine the factors affecting the development of pneumonia during the last four days. Generalized additive regression models were built to investigate effects of meteorological variables, gases, and lag effects on pneumonia admission. A *p* value smaller than 0.05 was accepted as statistically significant.

## 3. Results

A total of 2606 patients diagnosed with pneumonia were included in the study. The mean age of patients was 77.7 ± 7.6 years. Of the patients, 1387 (53.2%) were male and 1219 (46.8%) were women.

When the patients were included in the study according to CURB 65, the score of one (0.0) patient was found to be 0, score of 1116 (42.8%) patients was 1, score of 819 (31.4%) patients was 2, score of 377 (14.5%) patients was 3, score of 260 (10%) patients was 4, and score of 33 (1.3%) patients was found to be 5.

Of the patients, 1929 (74%) were discharged from the ER, 669 (25.7%) were hospitalized, 6 (0.2%) died in the ER, one (0.0) left the hospital at own request and one (0.0) was determined to be referred.

At least one patient admission was detected in 1227 (66%) of 1858 days taken into evaluation. The mean admission number during the days on which pneumonia patients were admitted was 2.1 ± 1.5 with the minimum admission number of one and maximum admission number of 14.

The mean temperature value on the day when the temperature was the highest was 17.5 ± 10.0°C, mean temperature value on the day when the temperature was normal was 10.12 ± 6.4°C, mean temperature value on the day when the temperature was the lowest was 4.4 ± 7.9°C while the mean humidity value on the day when the humidity was the highest was 87.7 ± 11.7%, mean humidity value on the day when the humidity was normal was 63.2 ± 16.9%, and mean humidity value on the day when the humidity was the lowest was 32.9 ± 20.9%. The mean pressure value when the sea-level pressure was the highest was 1019.2 ± 6.2 mmHg, mean pressure value when the sea-level pressure was normal was 1016.3 ± 5.6 mmHg, and, finally, when the sea-level pressure value was the lowest, the mean pressure value was 1012.7 ± 6.6 mmHg and wind speed was found to be 8.9 ± 4.3 per hour (Table 1).

Examining the air pollution parameters in our study, the mean PM 10 value was 62.2 ± 46.3, mean PM 2.5 value was 22.9 ± 18.2, mean SO_2_ value was 10.6 ± 8.1, mean NO value was 46.5 ± 46.6, mean NO_2_ value was 54.1 ± 21.9, mean NOX value was 101.2 ± 59.0, and mean CO value was found to be 34.7 ± 23.2 (Table 2).

During periods of high and low temperatures, the number of days admitted with pneumonia was significantly higher at low weather temperatures (*p* < 0.05). There was a negative correlation between the pneumonia and temperature. During the periods of normal temperatures, no relationship was detected between the number of days admitted with pneumonia and temperature (*p* > 0.05). During the periods of high, normal, and low humidity, the number of days admitted with pneumonia was higher at high humidity rates (*p* < 0.05). There was a positive correlation between the humidity and pneumonia. During the periods of high sea-level pressure, the number of days admitted with pneumonia was higher at high pressure levels (*p* < 0.05). There was also a positive correlation between the sea-level pressure and pneumonia. During periods when the sea-level pressure was normal and low, no significant relationship was detected between the number of days admitted with pneumonia and pressure levels (*p* > 0.05) (Table 3).

During the periods when PM 10, NO, NO_2_, NOX, and CO levels were high, the number of days admitted with pneumonia was detected to be increased (*p* < 0.05). There was a positive correlation between pneumonia and PM 10, NO, NO_2_, NOX, and CO levels. There was no significant relationship between the levels of PM 2.5 and SO_2_ and the number of days admitted with pneumonia (*p* > 0.05) (Table 4).

It was raining on 524 (42.7%) of the days admitted with pneumonia, but no rain was detected in 703 (57.3%) of them. It was raining during 218 (34.5%) of the days without pneumonia admission while no rain was detected during 413 (65.5%) of them. There was statistically significant relationship between the rainy days and admission with pneumonia (*p* < 0.05) (Table 5). There was a positive correlation between pneumonia and rain.

During the period of maximum temperature, it was determined that the temperature during the 4-day period before admission did not change the risk of admission with pneumonia (*p* > 0.05). During the period of mean temperature, it was determined that the temperature during the 4-day period before admission did not change the risk of admission with pneumonia (*p* > 0.05). In the period of minimum temperature, it was found that the temperature during the 4-day period before admission did not change the risk of admission with pneumonia (*p* > 0.05) while the risk of admission decreased as the temperature increased four days before admission (*p* < 0.05) (Table 6).

In the period of maximum humidity, it was detected that the humidity during the 4-day period before admission did not change the risk of admission with pneumonia (*p* > 0.05) and the risk of admission increased as the humidity increased two days before admission (*p* < 0.05). When the mean humidity rate was examined, it was found that the humidity in the 4-day period did not change the risk of admission with pneumonia (*p* > 0.05). During the period of minimum humidity, it was determined that the humidity in the 4-day period before admission did not change the risk of admission with pneumonia (*p* > 0.05) (Table 7).

During the period when the sea-level pressure was maximum, it was found that the pressure in the 4-day period before admission did not change the risk of admission with pneumonia (*p* > 0.05). During the period when the sea-level pressure was normal, it was determined that the pressure in the 4-day period before admission did not change the risk of admission with pneumonia (*p* > 0.05). During the period when the sea-level pressure was minimum, the pressure on days 0, 1, 3, and 4 before admission did not change the risk of admission with pneumonia (*p* > 0.05) and the risk of admission increased as the pressure on day 2 before admission increased (*p* < 0.05) (Table 8).

It was determined that the wind during the 4-day period before admission did not change the risk of admission with pneumonia (*p* > 0.05) (Table 9).

It was found that the levels of PM 10, PM 2.5, SO_2_, NO, and CO during the 4-day period before admission did not change the risk of admission with pneumonia (*p* > 0.05). It was determined that the level of NO_2_ during the 3-day period did not change the risk of admission with pneumonia (*p* > 0.05) whereas an increase was detected in the risk of admission as the level of NO_2_ increased (*p* < 0.05). It was determined that the level of NOX during the 3-day period did not change the risk of admission with pneumonia (*p* > 0.05) whereas an increase was detected in the risk of admission as the level of NOX increased (*p* < 0.05). There was no correlation between the period with high NOX level and three days before admission with pneumonia (*p* > 0.05) whereas a significant relationship was noted between the level of NOx on day 4 before admission and frequency of pneumonia (*p* < 0.05). On the day of admission, the elevation of CO increased the risk of admission (*p* < 0.05) while the risk of admission did not change during the remaining days (*p* > 0.05) (Table 10).

## 4. Discussion

Seasonal diseases are the pathologies occurring or worsening in weather conditions and seasonal variations [10]. Changes in meteorological factors usually affect the respiratory system. They cause especially bronchial asthma, tracheitis, pneumonia, and other respiratory tract pathologies [11]. Meteorological factors act by reducing the resistance of the human body to infection and by facilitating the spread of infection-causing pathogens [12, 13]. Pereira et al. reported that, despite a seasonal variation, the incidence of pneumonia varied as 1.9–12.7% [14].

Meteorological factors alter the permeability of thermal stress in the membranes and capillary resistance, render the skin vulnerable to infection by influencing the rate of sweating and evaporation, cause changes in the nasal mucosa, lead to hypoxia by causing vasoconstriction peripheral vessel, and impair body resistance by impeding ciliary movements in the respiratory tracts [13]. They increase virulence by prolonging the survival time of microorganisms and viruses and by influencing the spread of infectious agents in the atmosphere [15].

Many studies showed that the incidence of respiratory infection increased with the decrease in air temperature [16–18]. Liu et al. stated that there was a significant association between air temperature and the incidence of respiratory system diseases [19]. Gautret et al. expressed that the frequency of viral respiratory tract infections increased in cold weather [20]. Pica and Bouvier reported that viruses causing respiratory tract infections increase the risk of infection in cold weather [21]. Borges et al. stated that the frequency of the pneumonia caused by* M. catarrhalis* changed with the air temperature and increased during the winter months. The most commonly isolated microorganisms were found to be* S. pneumonia* and* H. influenza* [22]. Nascimento-Carvalho et al. identified the relationship of* M. catarrhalis* with temperature [23]. Jain et al. stated that the incidence of pneumococcal infection and rate of inpatients due to this reason increased during the winter months [24, 25]. Respiratory Syncytial Virus with lipid envelope followed by Influenza A virus was shown to be high [15]. It was reported that Influenza virus spread fast in cold and dry conditions whereas it was completely inactive at temperatures above 30°C [15]. The incidence of pneumonia was found to increase during the periods of low and high air temperatures and at low air temperatures in this study. There was a significant relationship between the lowest temperature of four days before and incidence of pneumonia. We believe that growth frequency and virulence of microorganisms are increased and immune systems of the patients are weakened due to decreasing temperature. We suggest that the factors such as the mucociliary movement decreased secondary to cold and changes in membrane permeability increase the frequency of pneumonia. Also, we think that the onset of pneumonia lasts for four days since microorganisms have an incubation period.

Liu et al. showed that low humidity increased the development of infectious respiratory tract diseases and that it had a positive correlation with the number of hospitalization due to lower respiratory tract infection [19]. A study on the correlation between climatic factors including humidity and clinical diseases revealed that humidity was negatively associated with the upper and lower respiratory tract infections [26]. Borges et al. stated that the frequency of pneumonia caused by* M. catarrhalis* increased with humidity [22]. Nascimento-Carvalho et al. identified the relationship of* M. catarrhalis* with humidity [23]. Enveloped viruses such as influenza virus require 20–30% humidity for growth and they can only survive in weather with high humidity (50–70%) [15]. In our study, the frequency of pneumonia was determined to increase during periods and weather conditions with high humidity. A relationship was detected between the highest humidity rate of two days before and frequency of pneumonia. Although increasing humidity is suitable for the growth of some microorganisms, we suggest that some microorganisms grow more comfortably at high humidity rates. We also believe that increasing humidity will destroy the mucociliary activity in the respiratory system and create a favorable environment for microorganisms. We suggest that microorganisms do not produce infection even in a suitable environment before an incubation period is over and that this period of time is two days when the environment is suitable.

Shaman and Kohn stated that virus spread and survival were easier in the regions with low pressure [27]. Yang et al. emphasize that people should be careful about respiratory tract infections during hot and humid days in the low tropical regions where the pressure is low [28]. Liu et al. showed that the atmospheric pressure had a positive correlation with the number of hospitalizations due to lower respiratory tract infection [19]. The frequency of pneumonia was determined to be increased when the sea-level pressure was the highest and at high pressure levels. A relationship was found between the lowest pressure level before two days and frequency of pneumonia. We suggest that the activity of microorganisms and reaction of the body change depending on the humidity and temperature at increasing pressure levels.

In the study by Du Prel et al., although not as much as humidity and air temperature, rainy weather was reported to change the frequency of pneumonia [29]. Liu et al. stated that the amount of precipitation was associated with pneumonia [19]. The frequency of pneumonia was found to be increased significantly on rainy days in our study. This may be due to the low air temperature on rainy days. We also believe that staying under rain can also increase the risk of infection.

Liu et al. expressed that the wind was not associated with pneumonia [19]. In our study, there was no significant relationship between wind and admission frequency in the 4-day period. The main reason for this may be that the wind does not have a long-term effect on the microorganism or individual.

Tuan et al. noted that the level of PM increased during the cold months and played an active role in the respiratory system pathologies [30]. Happo et al. identified that the response to infection decreased due to increased inflammatory activity and immunosuppressant effects with the emission of PM [31]. The studies on PM identified that particles causing air pollution show inflammatory and anti-inflammatory effects by adhering to the epithelium in the respiratory tract and alveoli and by impairing biological membranes and cellular barriers [31, 32]. In the study by Lin et al., PM 2.5 in air pollution was stated to increase both risk of infection and respiratory mortality [17]. In the same study, they reported that gases apart from PM (NO_2_, SO_2_, and O_3_) affected respiratory infections merely [17]. Lin et al. also showed that pulmonary mortality was high on days when PM 10 levels were high [33]. Another study by Lin et al. in China's Pearl River region reported a significant association between the increase in PM 2.5 levels and respiratory mortality [34]. Zhao and colleagues found that PM 2.5 and PMc (particles between PM 2.5 and PM 10) were significantly associated with respiratory diseases [35]. The frequency of respiratory diseases was stated to be increased in school children due to the low level of air circulation in classrooms [36]. In our study, the frequency of pneumonia was determined to increase as the number of PM increased, but the difference was found to be significant for PM 10. There was no significant relationship between PM level in the past days and frequency of pneumonia. We suggest that increasing particles weakens the immune system and let microorganisms settle more easily by adhering to the epithelium and by causing impairment in the mucociliary activity and biological membranes. We believe that the result that there was no significant relationship between the past days and pneumonia was due to the fact that PM measurements do not change frequently.

Tuan et al. stated that SO_2_ increased the frequency of respiratory tract infections by reaching peak concentrations during the cold months [30]. Lin et al. reported that SO_2_ level affected respiratory tract infections at an insignificant level [17]. No significant relationship was detected between SO_2_ level and pneumonia as well as between SO_2_ level in the past days and pneumonia in our study. Although the growth of some microorganisms increases in the presence of SO_2_, we suggest that it does not have any effect on the presence of pneumonia since the level of SO_2_ found in the nature is very low.

Substances such as NO, NO_2_, and NOX are the chemical substances which are produced by the toxic gases, coming especially from vehicle exhausts and leading to the formation of ozone gas [30]. Ozone, a strong oxidant pollutant, increases the susceptibility to infection and inflammation in the respiratory tract [36–39]. The study by Chiu et al. stated that exposure to ozone played a role in the admission to hospitals with pneumonia during both hot and cold days [40]. There are also studies indicating that ozone has no association with respiratory tract infections [9, 41]. Negrisoli and Nascimento determined that the frequency of pneumonia at urban level was related to ozone [42]. The frequency of pneumonia was significantly higher in the patients with high levels of NO_2_, NO, and NOX in our study. There was a significant relationship between the levels of NO and NOX before four days and frequency of pneumonia. We believe that the toxic gases formed cause damage to the cells of the respiratory tract by impairing the membrane structures of the cell, pump structures within the cell membrane, and energy system, thus increasing the risk of infection. We suggest that this toxicity did not act without reaching to a certain level within the cell and that it takes about a period of four days since microorganisms cannot produce pneumonia before the incubation period is over.

Tuan et al. stated that the concentrations of CO increased during the winter months and caused infections [30]. Fusco et al. reported that, during the months when the concentration of CO was high, the frequency of asthma, COPD, and pneumonia was increased. The largest pollutant of respiratory tracts was stated to be CO [43]. Shvedova et al. argued that an effect similar to the toxic effect of carbon particles on the pharynx leads to pulmonary toxicity [44]. In our study, the frequency of pneumonia was significantly higher in patients with high CO level. The CO level in the past days was found to be associated with pneumonia. We believe that carbon monoxide damages cells by causing hypoxia especially at the tissue level and influencing the energy system in the cells, thereby increasing the susceptibility to infection. We suggest that toxicity is chronic and it is not influenced by the day, especially since the CO gas in the environment does not go below a certain level during the winter months.

## 5. Limitations

Our study was conducted in a single center, which might limit the generalizability of our results. We also did not consider the day of the week and seasonal trends in the analysis.

## 6. Conclusion

In conclusion, the climatic (temperature, humidity, pressure levels, rain, etc.) and air pollution factors (airborne particles, CO, NO, and NOX) were found to be effective on the number of patients admitted with pneumonia.

## Figures and Tables

**Table 1:** Descriptive statistics of temperature parameters.

		Mean ± SD	Minimum–maximum
Temperature (°C)	High	17.6 ± 10.0	−8–39
	Average	10.1 ± 6.5	9,92–1033
	Low	4.4 ± 7.9	−24–20

Humidity (%)	High	87.7 ± 11.8	39–100
	Average	63.2 ± 16.9	22–100
	Low	32.9 ± 20.9	4–98

Sea level pressure (mmHg)	High	1019.2 ± 6.2	1000–1043
	Average	1016.3 ± 5.6	997–1035
	Low	1012.7 ± 6.6	992–1033

Wind speed (m/S)		8.9 ± 4.3	0–32

**Table 2:** Descriptive statistics for air pollution parameters.

	Mean ± SD	Minimum–maximum
PM 10 (*μ*g/m^3^)	62.2 ± 46.3	2–566
PM 2.5 (*μ*g/m^3^)	22.9 ± 18.2	2–175
SO_2_ (*μ*g/m^3^)	10.6 ± 8.1	0–103
NO (*μ*g/m^3^)	46.5 ± 46.6	2–358
NO_2_ (*μ*g/m^3^)	54.1 ± 21.9	7–131
NOX (*μ*g/m^3^)	101.2 ± 59.0	16–473
CO (*μ*g/m^3^)	34.7 ± 23.2	1–178

**Table 3:** Relationship between the pneumonia visits and temperature parameters.

		Pneumonia visits		*p*
		Present Mean ± SD	Absent Mean ± SD	
Temperature (°C)	High	16.1 ± 9.9	20.3 ± 9.7	**0.001**
	Average	1012.8 ± 6.8	1012.3 ± 5.9	0.109
	Low	3.2 ± 7.8	6.6 ± 7.6	**0.001**

Humidity (%)	High	88.6 ± 11.4	85.8 ± 12.1	**0.001**
	Average	64.9 ± 16.9	60 ± 16.6	**0.001**
	Low	34.2 ± 21.3	30.7 ± 20.2	**0.029**

Sea level pressure (mmHg)	High	1020 ± 6.5	1018.6 ± 5.3	**0.001**
	Average	1016.4 ± 5.9	1015.9 ± 5	0.061
	Low	1012.8 ± 6.8	1012.3 ± 5.9	0.109

**Table 4:** Relationship between pneumonia visits and air pollution parameters.

	Pneumonia visits		*p*
	Present Mean ± SD	Absent Mean ± SD	
PM 10 (*μ*g/m^3^)	63.8 ± 45.5	59.4 ± 47.8	**0.046**
PM 2.5 (*μ*g/m^3^)	23.4 ± 18.6	22.2 ± 17.3	0.629
SO_2_ (*μ*g/m^3^)	10.7 ± 8.1	10.5 ± 8	0.528
NO (*μ*g/m^3^)	51.3 ± 50.1	37.5 ± 37.7	**0.001**
NO_2_ (*μ*g/m^3^)	55.2 ± 22.5	51.9 ± 20.7	**0.004**
NOX (*μ*g/m^3^)	107.2 ± 62.8	90.1 ± 49.4	**0.001**
CO (*μ*g/m^3^)	41.4 ± 24	31.3 ± 22	**0.001**

**Table 5:** Relationship between pneumonia visits and rain.

		Pneumonia visits		*p*
		Present *n* (%)	Absent *n* (%)	
Rain	Present	524 (42.7)	218 (34.5)	**<0.001**
	Absent	703 (57.3)	413 (65.5)	

**Table 6:** Temperature for 4 days before visit due to pneumonia.

	Day 0 OR (Min–Max)	1 day before visit OR (Min–Max)	2 days before visit OR (Min–Max)	3 days before visit OR (Min–Max)	4 days before visit OR (Min–Max)
Maximum (°C)	0.971 (0.937–1.006)	1.038 (0.989–1.089)	0.994 (0.947–1.043)	0.973 (0.927–1.021)	0.982 (0.948–1.018)
*p*	0.107	0.13	0.799	0.26	0.32
Average (°C)	0.988 (0.941–1.037)	1.021 (0.954–1.093)	0.999 (0.934–1.07)	0.972 (0.909–1.04)	0.969 (0.924–1.017)
*p*	0.624	0.542	0.985	0.41	0.205
Minimum (°C)	1.012 (0.976–1.049)	0.984 (0.941–1.029)	0.989 (0.945–1.034)	0.996 (0.952–1.041)	0.962 (0.928–0.997)
*p*	0.52	0.474	0.619	0.853	0.036

**Table 7:** Humidity for 4 days before visit due to pneumonia.* *

	Day 0 OR (Min–Max)	1 day before visit OR (Min–Max)	2 days before visit OR (Min–Max)	3 days before visit OR (Min–Max)	4 days before visit OR (Min–Max)
Maximum (%)	1.011 (0.998–1.025)	0.993 (0.978–1.008)	1.016 (1.001–1.032)	1.003 (0.988–1.018)	1.005 (0.992–1.019)
*p*	0.09	0.363	**0.038**	0.714	0.423
Average (%)	1.006 (0.993–1.018)	0.997 (0.982–1.013)	1.003 (0.987–1.019)	1.008 (0.992–1.024)	1.006 (0.994–1.019)
*p*	0.371	0.736	0.719	0.321	0.32
Minimum (%)	1.004 (0.996–1.012)	0.997 (0.988–1.006)	1.001 (0.992–1.011)	1.005 (0.996–1.014)	1.003 (0.995–1.011)
*p*	0.275	0.501	0.789	0.305	0.483

**Table 8:** Sea level pressure for 4 days before visit due to pneumonia.

	Day 0 OR (Min–Max)	1 day before visit OR (Min–Max)	2 days before visit OR (Min–Max)	3 days before visit OR (Min–Max)	4 days before visit OR (Min–Max)
Maximum (mmHg)	1.027 (0.997–1.058)	0.991 (0.95–1.035)	1.038 (0.993–1.084)	0.99 (0.948–1.033)	1.018 (0.989–1.049)
*p*	0.074	0.689	0.099	0.641	0.223
Average (mmHg)	1.024 (0.993–1.056)	0.977 (0.933–1.023)	1.048 (0.999–1.100)	0.981 (0.937–1.027)	1.01 (0.979–1.041)
*p*	0.129	0.327	0.057	0.405	0.531
Minimum (mmHg)	1.024 (0.997–1.052)	0.971 (0.933–1.011)	1.044 (1.001–1.089)	0.988 (0.949–1.028)	1.002 (0.975–1.029)
*p*	0.085	0.149	**0.043**	0.554	0.885

**Table 9:** Wind speed for 4 days before visit due to pneumonia.

	Day 0 OR (Min–Max)	1 days before visit OR (Min–Max)	2 days before visit OR (Min–Max)	3 days before visit OR (Min–Max)	4 days before visit OR (Min–Max)
Wind speed (m/S)	0.997 (0.971–1.024)	0.998 (0.969–1.029)	0.979 (0.95–1.008)	0.992 (0.963–1.022)	0.995 (0.969–1.022)
*p*	0.825	0.921	0.153	0.588	0.721

**Table 10:** Air pollution parameters for 4 days before visit due to pneumonia.

	Day 0 OR (Min–Max)	1 days before visit OR (Min–Max)	2 days before visit OR (Min–Max)	3 days before visit OR (Min–Max)	4 days before visit OR (Min–Max)
PM 10 (*μ*g/m^3^)	1.001 (0.996–1.005)	0.999 (0.993–1.004)	1.001 (0.996–1.006)	0.999 (0.993–1.004)	1.002 (0.998–1.006)
*p*	0.799	0.654	0.727	0.646	0.300
PM 2.5 (*μ*g/m^3^)	1.004 (0.991–1.016)	1.002 (0.986–1.018)	1.002 (0.986–1.018)	1.008 (0.992–1.024)	0.997 (0.985–1.009)
*p*	0.562	0.801	0.801	0.345	0.623
SO_2_ (*μ*g/m^3^)	0.989 (0.966–1.011)	0.994 (0.968–1.02)	1.003 (0.976–1.032)	1.016 (0.987–1.047)	1.002 (0.978–1.027)
*p*	0.324	0.649	0.819	0.285	0.871
NO (*μ*g/m^3^)	1.001 (0.997–1.005)	1.002 (0.997–1.007)	1.004 (0.998–1.009)	1 (0.995–1.005)	1.004 (1–1.008)
*p*	0.609	0.51	0.179	0.897	0.066
NO_2_ (*μ*g/m^3^)	1.006 (0.997–1.015)	0.991 (0.979–1.003)	1.009 (0.996–1.021)	0.994 (0.982–1.006)	1.01 (1–1.019)
*p*	0.216	0.151	0.176	0.317	**0.040**
NOX (*μ*g/m^3^)	1.001 (0.998–1.005)	1 (0.996–1.004)	1.003 (0.999–1.007)	0.999 (0.995–1.003)	1.004 (1–1.007)
*p*	0.364	0.992	0.124	0.588	**0.026**
CO (*μ*g/m^3^)	0.985 (0.974–0.997)	1.005 (0.99–1.019)	0.994 (0.979–1.008)	1.004 (0.99–1.019)	0.99 (0.979–1.002)
*p*	**0.014**	0.538	0.388	0.585	0.105
